# *Haemoproteus minutus* is highly virulent for Australasian and South American parrots

**DOI:** 10.1186/s13071-018-3255-0

**Published:** 2019-01-17

**Authors:** Luis Ortiz-Catedral, Dianne Brunton, Mark F. Stidworthy, Hany M. Elsheikha, Tom Pennycott, Christoph Schulze, Michael Braun, Michael Wink, Helga Gerlach, Helene Pendl, Achim D. Gruber, John Ewen, Javier Pérez-Tris, Gediminas Valkiūnas, Philipp Olias

**Affiliations:** 1 Massey University, Institute of Natural and Mathematical Sciences, Massey University, Private Bag 102904, North Shore Mail Centre, Auckland, 0745 New Zealand; 2 International Zoo Veterinary Group, Station House, Parkwood Street, Keighley, BD21 4NQ UK; 3 University of Nottingham, School of Veterinary Medicine and Science, Sutton Bonington Campus, Sutton Bonington, Leicestershire, LE12 5RD UK; 4 Ayr Disease Surveillance Centre, Auchincruive, Ayr, KA6 5AE UK; 5 Berlin-Brandenburg State Laboratory, Gerhard-Neumann-Str. 2, 15236 Frankfurt (Oder), Germany; 6 Heidelberg University, Institute of Pharmacy and Molecular Biotechnology, Im Neuenheimer Feld 364, 69120 Heidelberg, Germany; 7 Gerlach Laboratory, Grosshessloher Strasse 23, 81479 Munich, Germany; 8 Pendl Laboratory, Untere Roostmatt 7, 6300 Zug, Switzerland; 9 Freie Universität Berlin, Institute of Veterinary Pathology, Robert-von-Ostertag-Str. 15, 14163 Berlin, Germany; 10 Zoological Society of London, Institute of Zoology, Regent’s Park, London, NW1 4RY UK; 11 Departamento de Biodiversidad, Ecología y Evolución, Facultad de Biología (Planta 9), Complutense University of Madrid, C/ José Antonio Novais, 2. Ciudad Universitaria, 28040 Madrid, Spain; 12 Institute of Ecology, Nature Research Centre, Akademijos str. 2, 08412 Vilnius, Lithuania; 13 University of Bern, Institute of Animal Pathology, Länggassstrasse 122, 3063 Bern, Switzerland

**Keywords:** *Haemoproteus*, *Plasmodium*, Malaria, Haemosporida, Apicomplexa, Psittaciformes, Parrot, Conservation

## Abstract

**Background:**

*Haemoproteus* and *Plasmodium* species are widespread avian blood parasites. Several *Plasmodium* species are known for their high virulence and have caused significant declines in naïve bird populations. The impact of closely related *Haemoproteus* parasites is largely unknown. Recently we reported a lethal disease in two parrot aviaries caused by *Haemoproteus* parasites.

**Results:**

Here we show that the causative pathogen *Haemoproteus minutus* is responsible for further 17 lethal outbreaks in parrot aviaries in Denmark, Germany and Great Britain. All affected parrots are endemic to Australasia and South America. We sequenced the cytochrome *b* gene from megalomeront-infected muscle tissue of 21 parrots and identified the two lineages TUPHI01 and TURDUS2 as causative agents, commonly naturally infecting the common blackbird (*Turdus merula*) and the song thrush (*Turdus philomelos*), respectively, in the Palaearctic. No intraerythrocytic parasite stages were found in any of the parrots. We failed to detect *H. minutus* in invasive Indian ring-necked parakeets (*Psittacula krameri*) in Germany. Together this suggests that abortive infections with two virulent lineages of *H. minutus* are lethal for naïve parrot species from Australasia and South America. We asked whether we could detect *H. minutus* in New Zealand, where its *Turdus* hosts were introduced in the 1800s. We therefore tested invasive blackbirds and song thrushes, and the co-existing endemic red-fronted parakeet (*Cyanoramphus novaezelandiae*) population on three New Zealand islands. No *Haemoproteus* spp. DNA was detected in all blood samples, indicating absence of transmission.

**Conclusions:**

The results of this study show that captive parrots in Europe are threatened by two lineages of an otherwise benign parasite of *Turdus* spp. Aviary collections of parrots should be protected from *Culicoides* spp. vectors in Europe. Animal trade and climate changes extending the current vector and parasite distribution have to be considered as potential risk factors for the introduction of the disease in naïve parrot populations.

## Background

Generalist pathogens (i.e. capable of infecting a wide range of hosts) can pose a serious threat for immunologically naïve animal host populations if they spread, e.g. by changing climate conditions, unintentional or deliberate introduction [1]. Emerging pathogens can have a devastating effect on the wildlife biodiversity as exemplified by the pandemic of the white nose syndrome fungus *Pseudogymnoascus destructans* in the USA responsible for severe bat population declines [2], and *Batrachochytrium* fungi causing widespread declines of amphibians worldwide [3, 4]. In the past decades, environmental changes such as global temperature rises accelerated the main driver of epidemics in wildlife, the human-assisted introduction of infectious agents into naïve ecosystems [5]. Often, novel pathogens do not cause obvious disease in evolutionary adapted host species of their origin and only become pathogenic in naïve host species [6]. However, the factors determining the pathogenicity potential at the molecular level, rendering some host species more susceptible than others are most often not well understood.

Another textbook example of an invasive pathogen spread is that of avian malaria on the islands of Hawaii in the late 19th century that occurred after the two-step introduction of (i) the mosquito vector *Culex quinquefasciatus* followed by (ii) the malaria parasite *Plasmodium relictum* [7]. Since then the native bird population has been devastated below 1200 m of elevation where the sole vector of *P. relictum* is most active [8]. Most notable are the major extinctions of the endemic bird population of Hawaiian honeycreepers (Fringillidae, subfamily Carduelinae) [7, 9]. *Plasmodium relictum* is the most successful malaria parasite worldwide, and is reported from over 300 avian species belonging to 11 orders [10]. Studies on experimentally infected birds found the *P. relictum* lineage GRW4 and the related species *P. elongatum* to be highly virulent for naïve hosts [11, 12]. In novel hosts these parasites cause a marked blood pathology including anemia and hemolysis caused by intraerythrocytic parasite stages. However, the tissue pathology caused by their exo-erythrocytic stages is less well understood [13, 14]. Avian malaria parasites of the genus *Plasmodium* are closely related to haemosporidian parasites of the genus *Haemoproteus*, which are also infecting erythrocytes among other cells of birds. We recently reported outbreaks of an emerging fatal disease in captive parrots (Psittaciformes) in Germany and Switzerland caused by *Haemoproteus* parasites [15]. Parasites of this genus are widespread and usually considered to be relatively benign in native songbirds [16]. Other than for *Plasmodium* spp., the negative impact of *Haemoproteus* spp. on naïve bird populations and its pathogenicity potential is less well studied [17]. However, recent reports suggest that *Haemoproteus* parasites are frequently causing fatalities in naïve bird species [14, 18].

In the present study, we genetically characterized the lineages of *Haemoproteus* species responsible for fatal disease outbreaks in captive parrots throughout Europe and recorded the phylogenetic range of susceptible parrot species. Exo-erythrocytic parasite stages and the associated host pathology are described in detail. Parrots (Psittaciformes) are now considered the most threatened bird order with 56% of all parrot species in decline [19], and 29% (115 of 399) species classified as vulnerable to critically endangered according the 2018 IUCN Red List [20]. Habitat loss and bird trade can be considered as main contributing threats [19]. Less is known on the impact of infectious diseases to this ongoing decline in parrot populations. Identifying causes of mortality and potential risk factors for infectious disease outbreaks are critical for the successful conservation management of highly endangered parrot species. We therefore tested the distribution of *Haemoproteus* parasites in free-ranging parrot populations and assessed the risk of a disease spread into vulnerable naïve parrot populations in New Zealand.

## Methods

Outbreaks of a fatal disease in parrots associated with megalomeronts of an unknown parasite species have been documented throughout Europe for a century [21]. We collected tissue samples of lung, heart, gizzard, proventriculus, kidney, spleen, liver and intestine from captive parrots that died during 17 outbreaks in Denmark, England, Germany, and Scotland between 2002 and 2011 (Table 1) and re-evaluated previously reported cases from the literature (Table 2). Formalin-fixed and paraffin-embedded or fresh tissue from heart and gizzard muscle infected with megalomeronts were used for subsequent molecular analysis. For histopathological analysis, representative tissues fixed in 4% phosphate-buffered formalin and paraffin-embedded, routinely sectioned at 4 μm and stained with haematoxylin and eosin (HE) were used. Imprints of unfixed tissue on glass slides were stained with Leishman stain.

**Table 1:** List of fatal cases due to *Haemoproteus minutus* infection in parrots analysed in this study

Case	Species	Native to	Year	*H. minutus* lineage	Country	Location	Coordinates
A645/06	Bourke’s parrot (*Neopsephotus bourkii*)	Australia	2006	TUPHI01	UK, Scotland	Ardbeg	55°51'25.0"N, 5°03'46.0"W
3536/1/10	Budgerigar (*Melopsittacus undulatus*)	Australia	2010	TUPHI01	UK, Scotland	Ardbeg	55°51'25.0"N, 5°03'46.0"W
8	Crimson rosella (*Platycercus elegans*)	Australia	2006	TURDUS2	Germany	Lübbenau	51°52'04.2"N, 13°58'07.5"E
257	Crimson rosella (*P. elegans*)	Australia	2009	TUPHI01	Germany	Horstdorf	51°49'03.7"N, 12°26'04.5"E
204	Crimson rosella (*P. elegans*)	Australia	2010	TUPHI01	Germany	Teichland	51°48'43.0"N, 14°25'22.3"E
06-0904	*Cyanoramphus* sp.	New Zealand	2006	TURDUS2	UK, England	Cheshire	NG
06-0822	Goldie’s lorikeet (*Psitteuteles goldiei*)	New Guinea	2006	TUPHI01	UK, England	West Yorkshire	NG
06-0845	Goldie’s lorikeet (*P. goldiei*)	New Guinea	2006	TURDUS2	UK, England	West Yorkshire	NG
10-1090	Monk parakeet (*Myiopsitta monachus*)	South America	2010	TURDUS2	UK, England	NG	NG
11-1259	Papuan lorikeet (*Charmosyna papou goliathina*)	New Guinea	2011	TURDUS2	UK, England	Harewood	53°53'39.6"N, 1°31'45.5"W
197	Turquoise parrot (*Neophema pulchella*)	Australia	2004	TURDUS2	Germany	Ruhland	51°27'22.0"N, 13°52'11.1"E
208	Turquoise parrot (*N. pulchella*)	Australia	2004	TUPHI01	Germany	Ruhland	51°27'22.0"N, 13°52'11.1"E
204	Red-fronted parakeet (*Cyanoramphus novaezelandiae*)	New Zealand	2005	TUPHI01	Germany	Melchow	52°46'33.0"N, 13°41'49.2"E
05-0739	Princess parrot (*Polytelis alexandrae*)	Australia	2005	TURDUS2	UK, England	West Yorkshire	NG
06-0903	Princess parrot (*P. alexandrae*)	Australia	2006	TUPHI01	UK, England	West Yorkshire	NG
173	Red-fronted parakeet (*C. novaezelandiae*)	New Zealand	2004	TUPHI01	Germany	Wandlitz	52°45'18.6"N, 13°28'22.6"E
129	Red-fronted parakeet (*C. novaezelandiae*)	New Zealand	2005	TUPHI01	Germany	Wandlitz	52°45'18.6"N, 13°28'22.6"E
209	Red-fronted parakeet (*C. novaezelandiae*)	New Zealand	2005	TUPHI01	Germany	Prenzlau	53°18'34.9"N, 13°51'46.1"E
2658/1/02	Superb parrot (*Polytelis swainsonii*)	Australia	2002	TUPHI01	UK, Scotland	Ardbeg	55°51'25.0"N, 5°03'46.0"W
11-1099	Superb parrot (*P. swainsonii*)	Australia	2011	TUPHI01	UK, England	Harewood	53°53'39.6"N, 1°31'45.5"W
08-524	Western rosella *Platycercus icterotis*)	Australia	2008	TUPHI01	Denmark	Hårlev	55°21'01.6"N, 12°13'37.6"E

*Abbreviation:* *NG* not given

**Table 2:** List of parrots described in the literature to be affected by megalomeront structures in the heart and gizzard muscle other than identified in this study

Species	Native to	Year	Country	Location	Reference
Red-winged parrot (*Aprosmictus erythropterus*)	Australia	1966	Germany	Mosbach	[55]
Scarlet-chested parrot (*Neophema splendida*)	Australia	1979	Germany	North Rhine-Westphalia	[56]
Horned parakeet (*Eunymphicus c. cornutus*)	New Caledonia	NG	Switzerland	NG	[57]
Northern rosella (*Platycercus venustus*)	Australia	1972	UK	NG	[58]
Mulga parrot (*Psephotus varius*)	Australia	1971	UK	NG	[59]
Pale-headed rosella (*Platycercus adscitus*)	Australia	1971	UK	NG	[59]
Australian king parrot (*Alisterus scapularis*)	Australia	1971	UK	Cornwall	[60]
Regent parrot (*Polytelis anthopeplus*)	Australia	1971	UK	Glos.	[60]
Yellow-crowned parakeets (*Cyanoramphus auriceps*)	New Zealand	2010	Switzerland	52°16'N, 13°39'E	[15]
Barred parakeet (*Bolborhynchus lineola*)	South America, Middle America	2010	Germany	47°25'N, 8°50'E	[15]

*Abbreviation:* *NG* not given

Furthermore, blood samples were taken from 183 free-ranging ring-necked parakeets (*Psittacula krameri*) of Asian origin in Heidelberg, Germany, from 2009 to 2011. Two urban breeding colonies (SHA, 49°25'03.5"N, 8°41'02.9"E; ST-WH, 49°25'08.7"N, 8°39'45.7"E) were chosen. The birds (153 nestlings and 30 adults) were caught in their nest boxes during the breeding season between May and August. About 100 μl of blood each bird was collected *via* brachial venipuncture and stored in EDTA buffer at -20 °C until DNA isolation with the QIAmp DNA Mini Kit (Qiagen, Hilden, Germany) following the manufacturer’s instructions.

Further blood samples were taken from two subspecies of free-ranging red-fronted parakeets (*Cyanoramphus novaezelandiae*; 138 adults and 59 nestlings) on three New Zealand islands in November 2009. Little Barrier Island (36°11'24.2"S, 175°04'32.2"E; *n* = 72 *C. n. novaezelandiae* sampled) and Tiritiri Matangi Island (36°36'01.0"S, 174°53'21.7"E; *n* = 37 *C. n. novaezelandiae*) are located in close proximity to the Northern main island in the Hauraki gulf, whereas Raoul Island (29°15'48.4"S, 177°55'46.2"W, *n* = 88 *C. n. cyanurus*) is located ~ 995 km northeast off the coastline of the Northern main island. Little Barrier Island is an island of 3083 ha with the most pristine native avifauna remaining in New Zealand. Tiritiri Matangi Island is a smaller 220 ha-island on which most extant native avian species have been reintroduced over the past decades. Raoul Island (2938 ha) is a remote volcanic island on which breeding of *C. n. cyanurus* was recorded again in 2008 after an absence for 172 years. Most likely parakeets recolonised the island from the Herald Islets after goats, rats and cats have been removed [22]. In all locations, adult parakeets were captured using mist-nets, whereas nestlings were taken from their nests. From each parakeet about 70 μl of blood was collected *via* brachial venipuncture using a heparinised capillary tube and stored at 4 °C until DNA extraction two days later with the Extract-N-Amp Tissue Kit (Sigma-Aldrich, St. Louis, USA) following the manufacturer’s instructions. Blood samples from common blackbirds (*Turdus merula*; *n* = 44) and song thrushes (*Turdus philomelos*; *n* = 19) were taken from birds caught in mist nets on Little Barrier Island and Tiritiri Matangi Island in 2009. Both bird species are invasive species of European origin, which have been introduced in the late 19th century and firmly established themselves throughout New Zealand including Raoul Island [23]. About 70 μl of blood from each bird was collected *via* brachial venipuncture and stored in 100% ethanol until DNA extraction.

To detect the presence of haemosporidian DNA in muscle and blood samples, we used a nested polymerase chain reaction (PCR) amplification method described by Hellgren et al. [24]. This method amplifies a 479 bp segment of the parasite’s cytochrome *b* (*cytb*) gene. All PCR reactions were carried out using the GoTaq Flexi DNA Polymerase (Promega, Madison, Wisconsin, USA) according to the manufacturer’s instructions. The following PCR protocol was used for the amplification of parasite *cytb*: initial denaturation at 94 °C for 3 min, followed by 20 cycles at 94 °C for 30 s, 50 °C for 30 s, 72 °C for 45 s, and a final extension step at 72 °C for 10 min. For the nested reaction, the same PCR conditions were used with 35 cycles. Amplicons were resolved on a 2% agarose gel stained with ethidium bromide and photographed under UV light. The avian *cytb* gene was amplified as described [25] to control for the DNA quality of each blood sample. No template controls used to detect DNA contamination of the PCR reagents always tested negative. *Cytb* amplification products were purified using the NucleoSpin Extract II system (Macherey-Nagel, Düren, Germany) and sequenced by a commercial DNA sequencing service (Seqlab GmbH, Goettingen, Germany) using the same forward and reverse primers. Sequences were compared with all sequences listed in the GenBank and MalAvi (http://mbio-serv2.mbioekol.lu.se/Malavi/blast.html) databases using the BLAST program (https://blast.ncbi.nlm.nih.gov). Published *Haemoproteus cytb* sequences were derived from MalAvi database and trimmed to 351 bp and 479 bp length, respectively, for comparison by CLUSTALW implemented in MEGA6. Lineage codes and GenBank accession numbers are given below. SplitsTree v4.14 was used to calculate unrooted phylogenetic networks using the neighbour-net method.

## Results

**Fig. 1:**
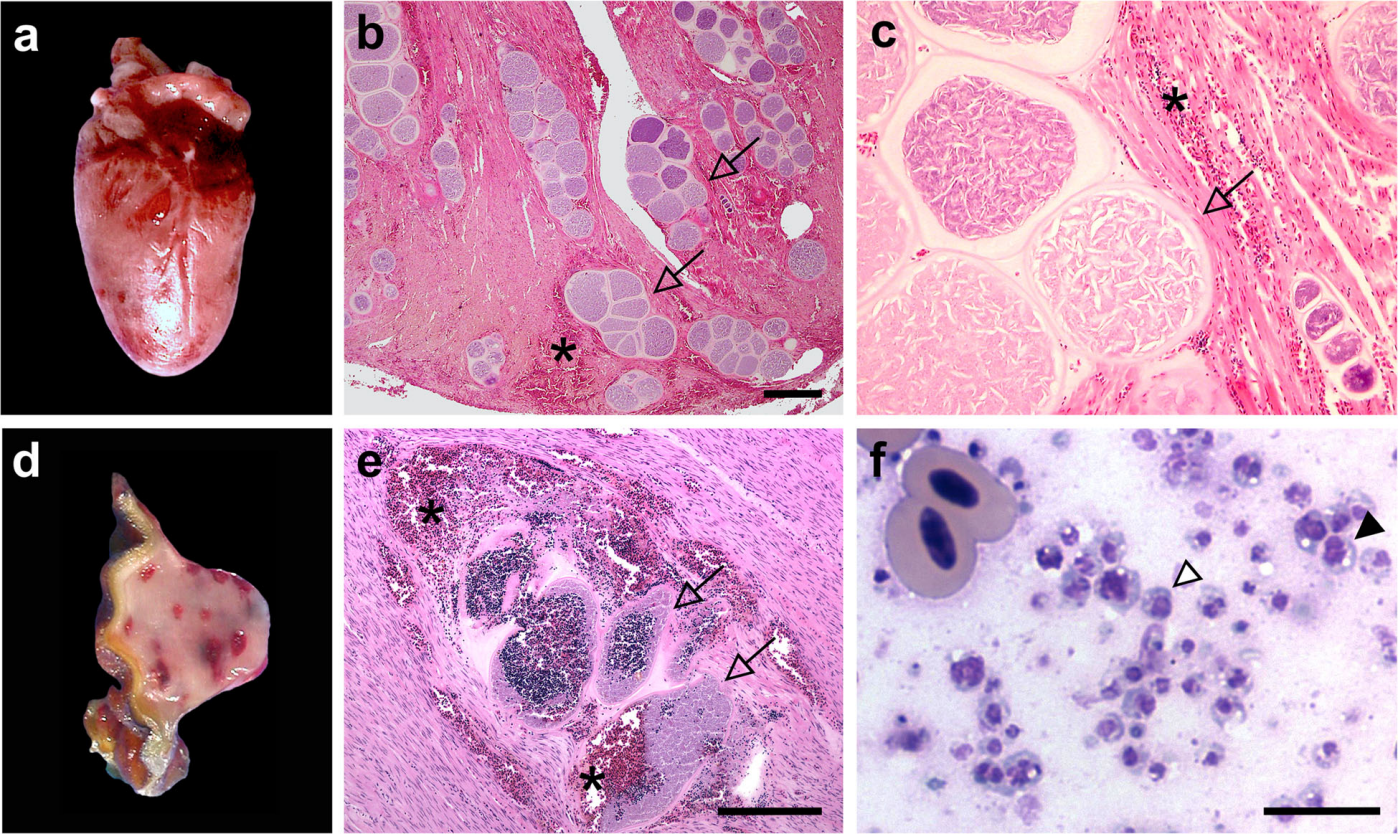
The heart and gizzard muscle of a two months old superb parrot (*Polytelis swainsonii*) that succumbed to disease showed severe infection with megalomeronts (arrows) of *Haemoproteus minutus*. Multifocally extended haemorrhages are readily visible in the heart muscle (**a**) and gizzard muscle (**d**) associated with megalomeronts (**e**) in this parrot. Numerous roundish megalomeronts in different stages of growth (**b**), each covered with a prominent capsular-like wall (**c**, which is an insert of **b**) disrupt the architecture of the heart muscle in a two weeks old Princess parrot (*Polytelis alexandrae*). Note the lack of a cellular host immune response towards the parasite. Imprint of gizzard muscle haemorrhages (**f**) associated with disrupted megalomeronts; **e** shows mixture of erythrocytes with syncytia originated from ruptured megalomeronts (**f**). Each syncytium possesses a portion of the cytoplasm and one (white arrowhead) or several nuclei (black arrowhead). During abortive development, the syncytia are readily washed out to the circulation and provide templates for PCR amplification in the blood even if intraerythrocytic stages (gametocytes) are absent. Asterisks, host tissue hemorrhage. Haematoxylin & eosin (**b**, **c** and **e**) and Leishman (**f**) stain. *Scale-bars*: **b** and **e** 200 μm; **f** 10 μm

Large tissue cyst-like structures resembling *Haemoproteus* megalomeronts were detected in 21 parrots (Psittaciformes; Table 1). Megalomeronts were located in the heart and gizzard muscle and frequently associated with extensive haemorrhages (Fig. 1a-e). Occasionally, megalomeronts were also found in other organs such as the lung (not shown). Macroscopically, parasite-associated haemorrhages appeared randomly scattered throughout the muscle tissue (Fig. 1a, d). Parasite structures were visible only microscopically (Fig. 1b, c, e). No host immune cell reaction was observed against the parasite. Megalomeronts were very often disrupted, and the remnants of the parasites (syncytia containing a portion of the cytoplasm and one or several nuclei) were present mixed with erythrocytes (Fig. 1f). Notably, we failed to identify intraerythrocytic *Haemoproteus* stages (gametocytes) in all parrots by histopathology. Although analysing erythrocytes in histopathological tissues sections is less reliable than in blood smears, this finding suggests that *H. minutus* does not complete its life-cycle and aborts the development of tissue stages in exotic parrot species.

Of the 21 individual parrots in which megalomeronts were detected in this study, 20 were native to Australasia and one to South America (Table 1). Phylogenetically, most species belonged to the tribes Platycercini and Psittaculini within the subfamily Psittacinae [26] (Table 1). *Haemoproteus minutus* lineages TURDUS2 and TUPHI01 were the only two parasite lineages detected by the comparison of amplified *cytb* gene sequences from infected muscle tissue samples with publicly available sequences (MalAvi database, http://mbio-serv2.mbioekol.lu.se/Malavi/blast.html; Table 1). A literature search for reports morphologically describing cysts-like structures resembling exo-erythrocytic megalomeront stages of *H. minutus* identified further eight parrots of the Platycerini and Psittaculini tribes native to Australasia as potentially susceptible species (Table 2).

**Fig. 2:**
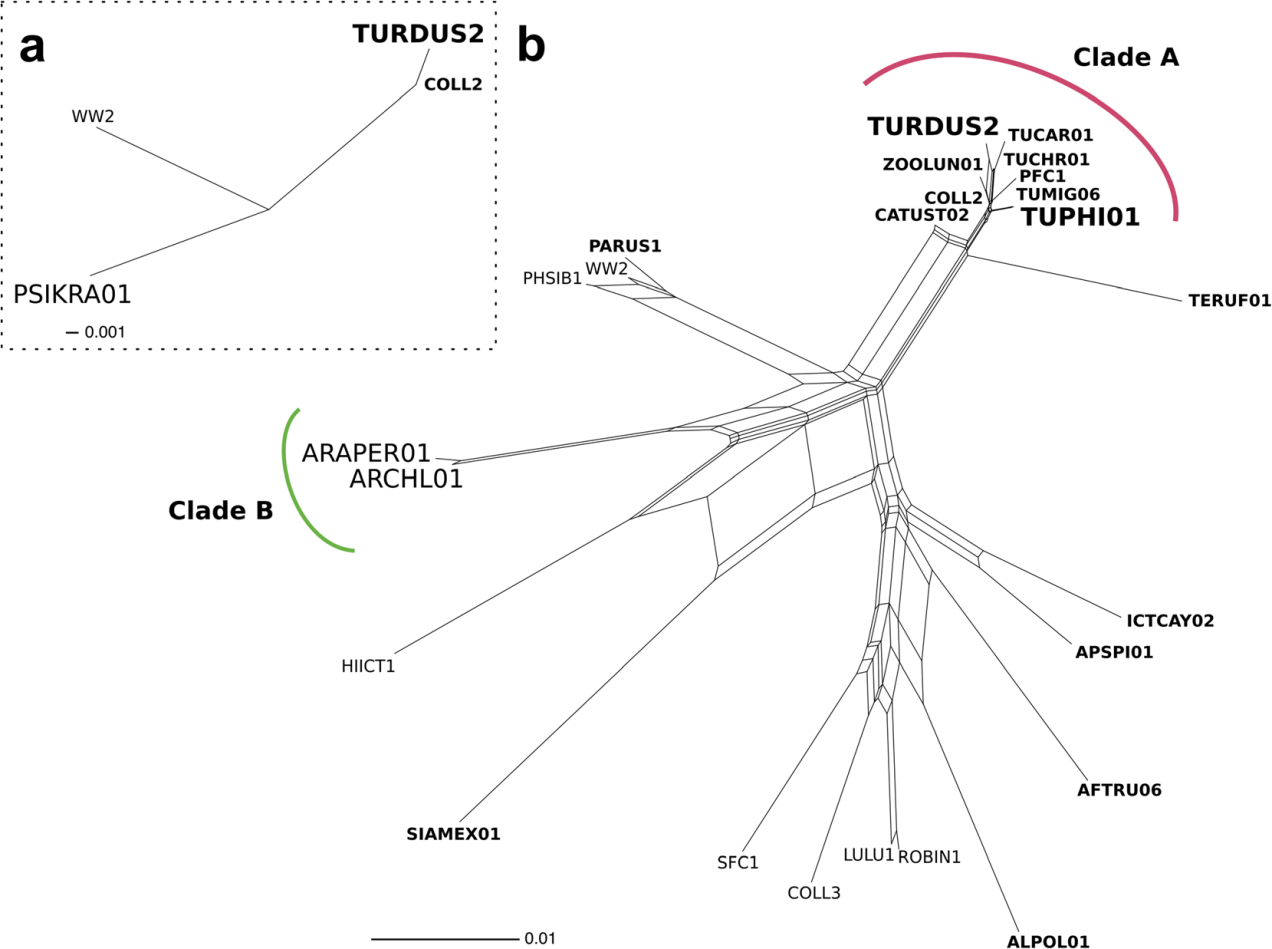
Cytochrome *b* sequences were incorporated into SplitsTree v4.14 to calculate unrooted phylogenetic networks using a neighbour-net method with 1000 bootstrap replicates. **a** Generalist TURDUS2 lineage of *H. minutus* is closely related to lineage COLL2 of *H. pallidus* which also has a wide host spectrum and geographical distribution, and is only distantly related to the lineage PSIKRA01 from the ring-necked parakeet (*P. krameri*). **b** *H. minutus* (lineages TURDUS2, TUPHI01 and TUCHR01) cluster with lineages from thrushes (Turdidae; clade A) and are only distantly related to known parrot (Psittaciformes) lineages (clade B) from the brown-throated parakeet (*Eupistaula pertinax*; ARAPER01) and green-winged macaw (*Ara chloropterus*; ARCHL01). Lineages reported from thrushes are shown in bold. Cytochrome *b* sequences were derived from MalAvi and GenBank database and were trimmed for comparison to 351 (**a**) and 479 nucleotides (**b**), respectively. The following sequences were used: AFTRU06 (EU810734); ALPOL01 (AF465588); ARAPER01 (DQ241553); ARCHL01 (KY783725); ASPI01 (EF153652); CATUST02 (DQ490061); COLL2 (FJ355915); COLL3 (DQ067581); HIICT1 (JX026904); ICTAY02 (DQ241546); LULU1 (DQ060767); PARUS1 (AF254977); PFC1 (DQ63577); PHSIB1 (AF495565); PSIKRA01 (EF380207); ROBIN1 (AY393807); SFC1 (DQ060770); SIAMEX01 (AF465562); TERUF01 (EU819755); TUCAR01 (EF380166); TUCHR01 (EU676190); TUMIG06 (GQ141584); TUPHI01 (GU85191); TURDUS2 (DQ630013); WW2 (AY831755); ZOOLUN01 (AY714150)

In a phylogenetic analysis, we compared TURDUS2 and TUPHI01 *cytb* gene sequences to psittacine *Haemoproteus* lineages (Fig. 2a, b). Although gametocytes of *Haemoproteus* spp. have been reported from more than 17 parrot species [27, 28] (Robert Adlard, International Reference Centre for Avian Haematozoa, personal communication), indicating complete life-cycles in these avian hosts, the molecular data on *Haemoproteus* lineages infecting parrots is scarce. We therefore added the *cytb* gene sequence (GenBank: KY783725) derived from a blood smear positive for mature *Haemoproteus* gametocytes of a South American green-winged macaw (*Ara chloropterus*) to our analysis [29]. For the phylogenetic network construction, we also included *Haemoproteus* lineages reported to infect thrushes and birds of closely related host families. The results show that TURDUS2 and TUPHI01 cluster with lineages infecting thrushes (Turdidae; Clade A) and the generalist *H. pallidus* lineage COLL2, but are only distantly related to the parrot lineages ARAPER01, ARCHL01 and PSIKRA01 (Fig. 2).

Since our results suggest that parrot species from Australasia and to a lesser extent South America but not Asia and Africa can react highly sensitive to *H. minutus* infection and die from abortive megalomeront formation in the heart and other organs, we wondered whether wild parrot populations native to Asia and Africa are infected with *H. minutus*. We therefore chose to test blood samples of an invasive ring-necked parakeet (*Psittacula krameri*) population native to Asia, which habitat overlaps with an area where both virulent *H. minutus* strains are reportedly present [30]. We sampled 183 free-ranging parakeets (153 nestlings, 30 adults) caught in nest boxes from breeding colonies in Heidelberg, Germany. PCR for the avian *cytb* gene served as control for each sample. A comparison of the amplified *cytb* gene sequences with publicly available sequences in GenBank confirmed a most probable Asian origin of the parakeets [25]. Even though the ring-necked parrot in its native African-Asian range can become infected with *Haemoproteus* spp. [31], none of the 183 German birds tested positive for *Haemoproteus* spp. or *Plasmodium* spp.

Most exotic parrots affected by the disease in captivity in Europe are native to Australasia, with the exception of monk parakeets (*Myiopsitta monachus*) and barred parakeets (*Bolborhynchus lineola*) [15] (Table 2). We therefore decided to focus on Australasian species that share a distribution area with introduced *Turdus* thrushes and tested blood samples of native red-fronted parakeets (*C. novaezelandiae*) and introduced common blackbird and song thrush populations in New Zealand for the presence of *H. minutus* parasites. Red-fronted parakeets have been found to succumb to infection (Table 1) and can be considered at risk if *H. minutus* was introduced into the population. Blood samples were taken from 138 adult and 59 nestling parakeets from Little Barrier Island (*n* = 72), Tiritiri Matangi Island (*n* = 37) and Raoul Island (*n* = 88). Blood samples from common blackbirds (*n* = 44) and song thrushes (*n* = 19) were taken from birds caught in mist nets on Little Barrier Island and Tiritiri Matangi Island. Samples were processed as described above and *cytb* PCR amplicons were sequenced and compared to published sequences (MalAvi). We failed to detect *Haemoproteus* spp. infection in any of the parakeets or the thrushes. However, two *Plasmodium* lineages (GRW6, *n* = 3; CYNOV1, *n* = 17, GenBank: KY783726) were identified in the parakeets.

## Discussion

A growing number of reports suggests that *Haemoproteus* blood parasites are more virulent in naïve birds than formerly believed. The pathology caused by these parasites in novel avian hosts appears to be mainly attributed to the damage of internal organs by exo-erythrocytic megalomeront stages [14, 32]. Our genetic findings on megalomeronts in exotic parrots in Europe revealed the two lineages TURDUS2 and TUPHI01 of the species *H. minutus* as highly virulent for birds of several distant parrot families. The common blackbird (*Turdus merula*) and the song thrush (*Turdus philomelos*) are the natural host species for both parasite lineages in the Palaearctic [30]. The extensive development of abortive *H. minutus* megalomeronts in the myocardium associated with haemorrhages prompted us to conclude that the deaths in these infected parrots were most likely caused by fatal heart failure due to disruption of cardiac function. Intra-erythrocytic parasite stages (gametocytes) and associated anaemia or internal organ damage due to infected erythrocytes building up in small blood vessels were not found. However, although a cellular immune response towards the parasite stages was absent, a strong and potentially fatal systemic immune response against parasite and host cell debris as well as toxic by-products cannot be excluded. It is also worth mentioning that deaths due to *Haemoproteus* infections have been reported in Asian and African parrots associated with intra-erythrocytic parasite stages [33–35], but pathological mechanisms underlying the mortality remained unclear. Experimental studies on *H. minutus* would be needed to better understand the disease mechanisms and the pathogenic potential of the two virulent parasite lineages TURDUS2 and TUPHI1 in birds. Sporogonic development of *H. minutus* isolated from the common blackbird in *Culicoides impunctatus* and *Culicoides nubeculosus* has recently been successfully achieved [17, 36]. This work opened new opportunities to study the host-parasite interaction of *H. minutus* in the context of natural and aberrant host species on the molecular level.

Naïve parrot populations can be considered at risk, if virulent *H. minutus* lineages get introduced and established in a habitat that overlaps with an invasive thrush population where susceptible *Culicoides* vectors are available. Of the parrot species that we found to be susceptible to *Haemoproteus* spp. in this study, 95% (18/19) are endemic to Australasia and one species to South America. Australasia and South America harbour the highest parrot diversity of extant species and the highest density of threatened bird species [37]. This great diversity of parrots in tropical and subtropical ecosystems of the southern hemisphere is markedly understudied in terms of their parasite diversity and only few parasites and their lineages have been described so far [29]. In addition to this study and a recent case report [15], the *H. minutus* lineage TUPHI01 has so far only been detected in wild thrushes (Turdidae) in Europe and eastern Russia. In contrast, the lineage TURDUS2 can be considered a generalist (multi-host) lineage, which was found in nine avian families distributed throughout the Holarctic (MalAvi). However, it remains unclear if the parasite completes its life-cycle in all of these birds since previous studies have shown that DNA from remnants of exo-erythrocytic stages of *Haemoproteus* species can be detected by PCR in the blood of infected birds [14, 15, 38]. Thrushes endemic to the Holarctic are considered adapted type-hosts of *H. minutus*, in which the parasite does not cause overt disease [17, 29]. Generalist blood-borne parasites that are able to infect multiple host species have been shown to be efficient colonisers of naïve avifaunas [39]. In Europe, the biting midge *Culicoides impunctatus* transmits *H. minutus* [17], but at least five other *Culicoides* spp. may also be involved in transmission (MalAvi). Therefore, the generalist *H. minutus* lineage TURDUS2 must be considered the most notable candidate for a disease spread if the distribution area of *H. minutus* and appropriate *Culicoides* insect vectors shift southwards.

Our findings show that parrots from Australasia and South America develop disease, while parrots native to Asia and Africa (part of the endemic range of *H. minutus*) appear to be refractory. Interestingly, blood samples taken from free-ranging ring-necked parakeets in Heidelberg, Germany, a region where both *H. minutus* lineages are present [40, 41], tested negative for *Haemoproteus* and *Plasmodium* parasite DNA. Ring-necked parakeets are an invasive species introduced to Germany since the 1960s and are resident in the Rhine-Neckar-region since 1974 [42]. Based on previous and our phylogenetic analysis of the *cytb* gene, the birds sampled from Heidelberg are of Asian origin [43]. As a very successful invasive species they have established multiple breeding colonies in urban areas in several European countries. In Asia, ring-necked parakeets have been reported to be infected with the *Haemoproteus* lineage PSIKRA01 [44]. We therefore concluded that the Asian *P. krameri* subspecies most likely co-evolved with a host-specific *Haemoproteus* lineage and is refractory to infections by *H. minutus*, probably due to their long-time overlapping distribution areas. Notably, a recent study suggests that some wild parrot species have a lower prevalence of haemoparasites than other birds due to the ingestion of secondary metabolites from their natural plant food which potentially function as antimalarials [45]. This could be another explanation why we did not detect any haemosporidian DNA in these free-living parakeets.

*Haemoproteus minutus* has not yet been found in molecular studies of avian species endemic to the southern hemisphere (MalAvi), although records from New Zealand from the early 20th century on the morphology of blood parasites from invasive common blackbirds and song thrushes suggest different [46]. We therefore decided to test invasive common blackbirds and song thrushes in New Zealand for *Haemoproteus* infection. We also sampled endemic and highly susceptible red fronted parakeets (*C. novalaezandiae*; Table 1) populations in overlapping island habitats. Surprisingly, we failed to detect *Haemoproteus* parasites in any of these birds. This result might be explained by the absence of appropriate insect vectors in New Zealand, which is currently considered free of *Culicoides* midges (Ministry of Primary Industries, New Zealand; https://www.mpi.govt.nz/protection-and-response/finding-and-reporting-pests-and-diseases/surveillance-programmes/). Consequently, it is likely that *Haemoproteus* parasites originally introduced with European thrushes were lost due to the current absence of suitable vectors.

New Zealand has one of the highest alien bird species richness [47] and invasive species have been identified as single most prevalent threat to parrots in Australasia [48]. Invasive thrushes have been suggested to serve as important reservoir for avian *Plasmodium* parasites with potential spill-overs of virulent lineages in native bird populations [49]. Our data suggest, that other than for avian *Plasmodium* spp., which are transmitted by endemic mosquitos, a two-step introduction of (i) a suitable vector and (ii) a virulent *H. minutus* lineage would currently be needed to establish the full infection circle in thrushes and transmissions into naïve *Cyanoramphus* parrot populations in New Zealand. In contrast, more than 200 biting midges have been described from Australia and are potentially serving in the transmission of > 60 *Haemoproteus* parasite lineages reported from Australian birds so far [50] (MalAvi). The only genetically characterized lineage of a thrush-infecting *Haemoproteus* spp. in Australia has been reported from the Bassian thrush (*Zoothera lunulata*) [51]. This thrush lineage, named ZOOLUN01 is closely related to TURDUS2 and COLL2 (*Haemoproteus pallidus*; Fig. 2b), which was isolated from a tooth-billed bowerbird (*Scenopoeetes dentirostris*) in Australia. COLL2 is infecting six bird families on four continents and as TURDUS2 can be considered a generalist *Haemoproteus* lineage (MalAvi). In the States of Victoria and New South Wales parrot habitats overlap with invasive European thrushes [52]. It is therefore tempting to speculate that other than for New Zealand, the introduction of *H. minutus* parasites into these thrush populations could be sufficient to cause die-offs in vulnerable parrot populations of this area.

## Conclusions

We identified 18 Australasian and one South American parrot species susceptible to fatal *H. minutus* infections. To avoid mortality, these birds should be protected from bites of *Culicoides* spp. vectors in captivity in Europe. Multi-host (generalist) parasites such as the virulent *H. minutus* lineage TURDUS2 have to be considered suitable invaders of naïve avian ecosystems [53] by virtue of having a more diverse array of susceptible definitive hosts (i.e. biting midges) and intermediate host (bird) species [54]. Single-country endemism is one important predictor of extinction risk for Australasian parrots [48]. Further studies are needed to assess the importance of potential other risk factors for extinction events including the introduction of invasive blood parasites and the contribution of e.g. commercial animal trade, bird migration, and global climate change.

## Abbreviations

DNA: Deoxyribonucleic acid
EDTA: Ethylenediaminetetraacetic acid
PCR: Polymerase chain reaction
